# Trends of ‘urolithiasis: interventions, simulation, and laser technology’ over the last 16 years (2000–2015) as published in the literature (PubMed): a systematic review from European section of Uro-technology (ESUT)

**DOI:** 10.1007/s00345-017-2055-z

**Published:** 2017-06-07

**Authors:** Amelia Pietropaolo, Silvia Proietti, Rob Geraghty, Andreas Skolarikos, Athanasios Papatsoris, Evangelos Liatsikos, Bhaskar K. Somani

**Affiliations:** 1 University Hospital Southampton NHS Trust, Southampton, SO16 6YD UK; 2 Raffaele Hospital, Ville Turro Division, Milan, Italy; 3 National and Kapodistrian, University of Athens, Athens, Greece; 4 Department of Urology, University of Athens, Sismanoglio General Hospital, Athens, Greece; 5 European Section of Uro-Technology (ESUT), Athens, Greece; 6 Patras University, Patras, Greece

**Keywords:** Publication, Trends, Urolithiasis, Stone, Ureteroscopy, Simulation, Laser

## Abstract

**Purpose:**

To look at the bibliometric publication trends on ‘Urolithiasis’ and aspects of treatment and training associated with it over a period of 16 years from 2000 to 2015. To this end, we conducted this study to look at the publication trends associated with urolithiasis, including the use of simulation, laser technology, and all types of interventions for it.

**Materials and methods:**

We performed a systematic review of the literature using PubMed over the last 16 years, from January 2000 to December 2015 for all published papers on ‘Urolithiasis’. While there were no language restrictions, English language articles and all non-English language papers with published English abstracts were also included. Case reports, animal and laboratory studies, and those studies that did not have a published abstract were excluded from our analysis. We also analyzed the data in two time periods, period-1 (2000–2007) and period-2 (2008–2015).

**Results:**

During the last 16 years, a total of 5343 papers were published on ‘Urolithiasis’, including 4787 in English language and 556 in non-English language. This included papers on URS (*n* = 1200), PCNL (*n* = 1715), SWL (*n* = 887), open stone surgery (*n* = 87), laparoscopic stone surgery (*n* = 209), pyelolithotomy (*n* = 35), simulation in Endourology (*n* = 82), and use of laser for stone surgery (*n* = 406). When comparing the two time periods, during period 2, the change was +171% (*p* = 0.007), +279% (*p* < 0.001), and −17% (*p* = 0.2) for URS, PCNL, and SWL, respectively. While there was a rise in laparoscopic surgery (+116%), it decreased for open stone surgery (−11%) and pyelolithotomy (−47%). A total of 82 papers have been published on simulation for stone surgery including 48 papers for URS (67% rise in period-2, *p* = 0.007), and 34 papers for PCNL (480% rise in period-2, *p* < 0.001). A rising trend for the use of laser was also seen in period 2 (increase of 126%, *p* < 0.02, from 124 papers to 281 papers).

**Conclusions:**

Published papers on intervention for Urolithiasis have risen over the last 16 years. While there has been a steep rise of URS and minimally invasive PCNL techniques, SWL and open surgery have shown a slight decline over this period. A similar increase has also been seen for the use of simulation and lasers in Endourology.

## Introduction

Urolithiasis is a major clinical and economic burden for modern healthcare systems. International epidemiological data suggest that the prevalence of kidney stone disease (KSD) is increasing, with a lifetime prevalence of around 14% [1, 2] and a recurrence rate of 50% or more within 10 years [3]. Although the mean age of patients with KSD is between 40 and 60 years, there is an alarming increase in the number of children diagnosed with stone disease [1, 3].

This rise in the stone disease is multifactorial with warm weather, dehydration, and metabolic syndrome all contributing to it [3–5]. Furthermore, technological advances and widespread use of imaging modalities have increased the likelihood of incidental stones [6]. The ever-increasing prevalence of urolithiasis directly affects the healthcare resources, with the trends of global ureteroscopy (URS) performed for stone disease increasing by 252% over the last two decades [2]. This emphasizes the importance of education and lifestyle adaptations in attempting to prevent stone formation.

The development of minimally invasive surgical techniques (MIS) for the treatment of patients suffering from KSD has been highly dependent on education and technological advances in the fields of simulation and laser technology. These advancements have accelerated the evolution of modern techniques of calculus removal, including URS, percutaneous nephrolithotomy (PNL), and shockwave lithotripsy (SWL), which have also fuelled the growth of publications in this field.

There is a growing burden of urolithiasis worldwide [1–6]. With global economic decline, health commissioners and clinicians need to justify their financial spending on urolithiasis-related research and intervention. Publication trends reflect changes of trends in clinical practice and this in turn leads to extra healthcare resource allocation, and clinical areas of importance are likely to attract more funding. Recent studies have shown a rise in the worldwide intervention for stone disease [2]; however ,there is no bibliometric study looking at the publication trends (2000–2015) on ‘Urolithiasis’ and aspects of treatment and training associated with it. To this end, we conducted this study to look at the publication trends associated with urolithiasis, including the use of simulation, laser technology, and all types of interventions for it.

## Materials and methods

**Fig. 1:**
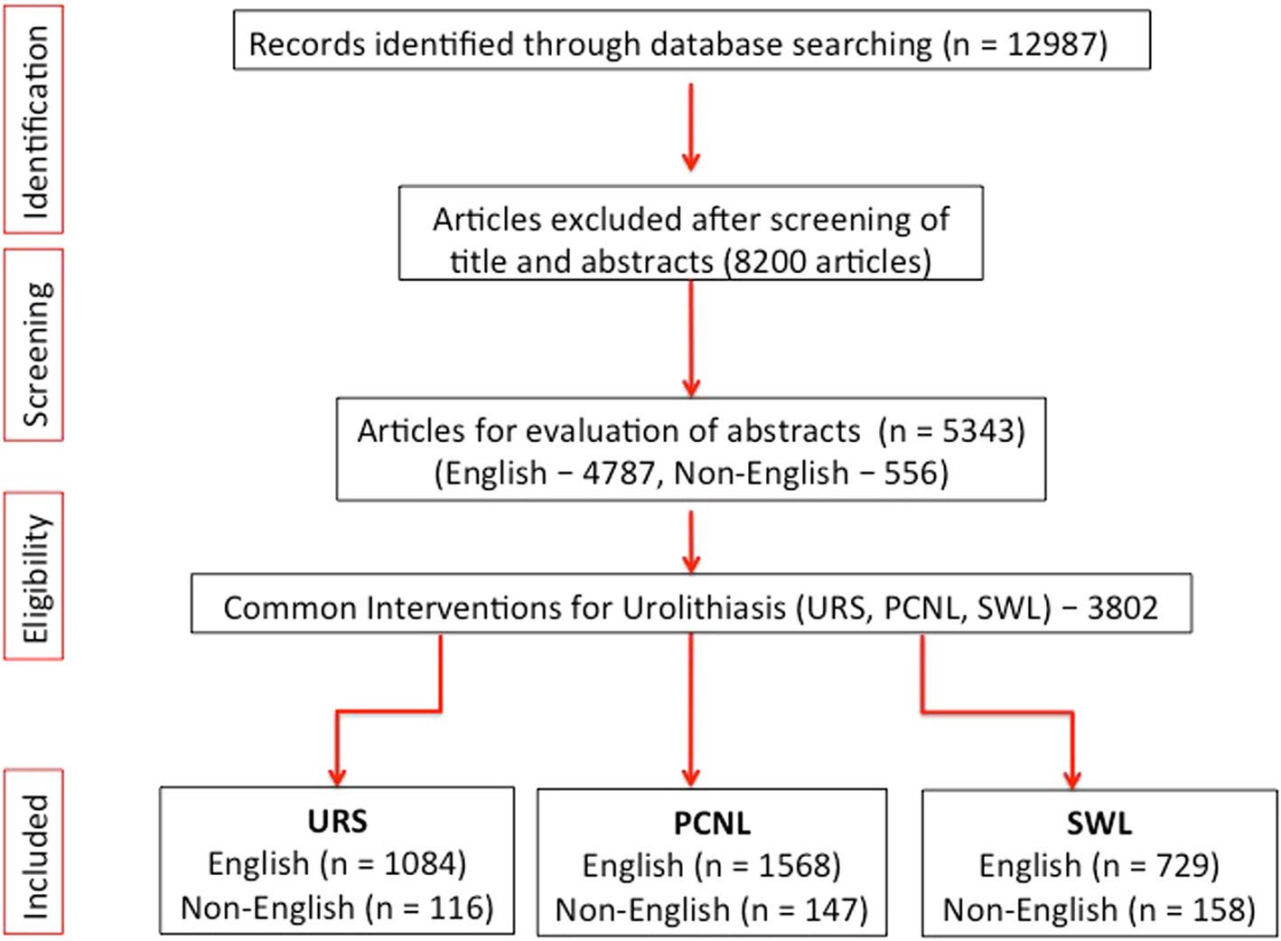
PRISMA flowchart for identification of the studies

 We conducted a systematic review of the literature using MeSH terms, title words, and key words in PubMed/MEDLINE over the last 16 years, from January 2000 to December 2015 for all published papers on ‘Urolithiasis’ (Fig. 1).

### Evidence acquisition: criteria for including studies for this review

#### Inclusion criteria

- All English language studies
- All non-English studies with abstracts written in the English language
- Studies reporting on urolithiasis: treatment and interventions—open surgery, laparoscopic surgery, pyelolithotomy, shockwave lithotripsy (SWL), percutaneous nephrolithotomy (PCNL), and ureteroscopy (URS),
- Studies on simulation in Endourology, and
- Studies on the use of laser technology in Endourology.

#### Exclusion criteria

- Studies without a published abstract
- Studies for non-urolithiasis conditions
- Animal and laboratory studies
- Case reports.

### Search strategy and study selection

The systematic review was performed according to the Cochrane Review and the preferred reporting items for systematic reviews and meta-analyses (PRISMA) guidelines. The search strategy was conducted to find all relevant abstracts regarding each specific topic, which was analyzed year by year from 2000 to 2015 (16 years). Specific terminology used was different for each topic. For URS, we examined all published papers on ‘Urolithiasis’, ‘ureteroscopy’, ‘URS’, ‘kidney stones’, ‘renal stones’, ‘ureteric stones’, ‘retrograde intrarenal stone surgery’, and ‘RIRS’. For PCNL, we considered ‘percutaneous nephrolithotomy’, ‘percutaneous stone surgery’, ‘PCNL’, and ‘PNL’. For SWL, we considered ‘shockwave lithotripsy’, ‘extracorporeal shockwave lithotripsy’, ‘SWL’, ‘ESWL’, and ‘lithotripsy’. For open stone surgery, we considered ‘open stone surgery’, for laparoscopy: ‘laparoscopic stone surgery’, ‘robotic surgery’, and ‘robotic stone surgery’ and for pyelolithotomy: ‘pyelolithotomy’.

For laser use in Endourology, we considered ‘laser’, ‘holmium’, ‘Ho:YAG lasers’, and ‘lasertripsy’. For simulation in Endourology, we considered ‘simulation’, ‘URS simulation’, ‘PCNL simulation’, ‘education’, ‘training’, ‘skills’, and ‘percutaneous access simulation’. These keywords were separately searched on PubMed among the literature published over the last 16 years from 2000 to 2015. Boolean operators (AND, OR) were used to refine the search. There were no language restrictions and all non-English language papers with published English abstracts were also included in our review but collected in a separate subgroup. While review articles were included, case reports and those studies that did not have a published abstract were excluded from our analysis. Similarly, animal and laboratory studies were discarded.

To make an effective comparison and to identify and contrast the different features, the data derived from each single research have been divided into two 8-year periods, period 1 (2000 to 2007) and period 2 (2008 to 2015). Data were collected using Microsoft Excel (version 2007) and analyzedusing the independent *t* test and Pearson’s correlation coefficient, using SPSS version 24.

## Results

### Overall number of papers on urolithiasis

**Fig. 2:**
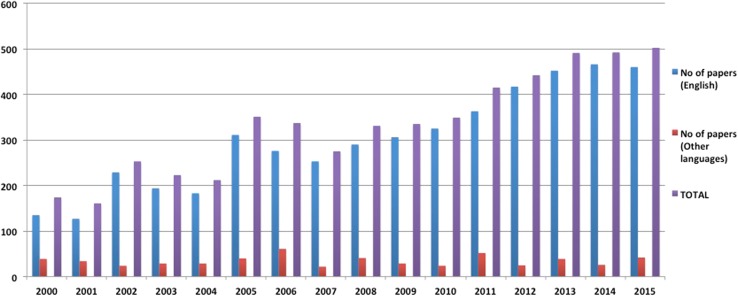
Publication trend for ‘Urolithiasis’: 2000–2015 (16-years)

During the last 16 years, a total of 5343 papers were published on ‘Urolithiasis’, including 4787 in English language and 556 in non-English language (Fig. 2; Table 1). When comparing the two time periods, while there were 1986 published papers in period 1, this had increased by 1.7 times to 3357 in period-2.

**Table 1:** All English and non-English language articles published over 16 years, 2000–2015

2000–2015	Urolithiasis (all)	URS	PCNL	SWL	Others
English	4787	1084	1668	729	1306
Non-English	556	116	147	158	135
French	132	36	27	26	43
Spanish	103	26	28	34	15
Chinese	68	12	39	11	6
German	68	21	24	23	0
Russian	66	7	16	31	12
Japanese	45	1	1	20	23
Italian	25	1	3	4	17
Polish	17	2	1	3	11
Romanian	9	3	2	1	3
Portuguese	9	2	0	1	6
Others	15	5	6	4	0

*URS* ureteroscopy, *PCNL* percutaneous nephrolithotomy, *SWL* shockwave lithotripsy

### Interventions for urolithiasis

**Fig. 3:**
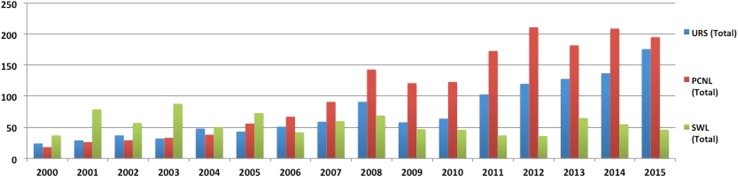
Publication trends for common interventions (URS, PCNL, SWL)

**Fig. 4:**
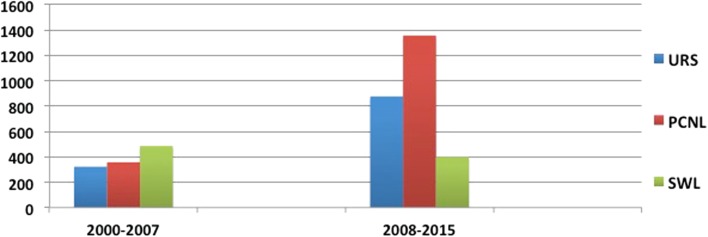
Publication trends for common interventions (URS, PCNL, SWL) over the two time periods (period 1: 2000–2007 and period-2: 2008–2015)

During the last 16 years, a total of 3802 papers have been published on common interventions for Urolithiasis, including URS (*n* = 1200, 32%), PCNL (*n* = 1715, 45%), and SWL (*n* = 887, 23%). When comparing the two time periods, there were 1167 intervention papers in period 1, which had more than doubled to 2635 intervention papers in period-2 (Figs. 3, 4). The increase was 171, 279, and −17% for URS, PCNL, and SWL, respectively.

#### Ureteroscopy (URS)

A total of 1200 URS papers were published on PubMed over these 16 years, of which majority (1084, 90%) were in English. Of the non-English articles (116, 10%), majority were in French (*n* = 38) and Spanish (*n* = 26).

There was a linear increase in the rates of URS over the study period (Fig. 3) for English language articles from 24 articles in 2000 to 176 articles in 2015 (*p* < 0.001). When comparing the two time periods, there were a total of 323 articles published in period 1, which had increased by × 2.7 times (171% rise) to 877 articles in period-2 (*p* = 0.001). The number of English/non-English language articles in period-1 and period-2 was 280/43 and 804/73 articles, respectively.

#### Percutaneous nephrolithotomy (PCNL)

A total of 1715 studies on PCNL were published on PubMed over a 16-year period [English language—1568 (91%) and non-English language—147 (9%)]. There was a linear increase in the rate of PCNL over the study period from 18 articles in 2000 to 195 articles in 2015 (*p* < 0.001) (Fig. 3). When comparing the two time periods, there were a total of 358 articles published in period 1, which had increased by almost four times (rise of 279%) to 1357 articles in period 2 (*p* < 0.001). The number of English/non-English language articles in period-1 and period-2 was 316/42 and 1252/105 articles, respectively.

#### Shockwave lithotripsy (SWL)

A total of 887 studies on SWL were published on PubMed over a16-year period [English language—729 (82%) and non-English language—158 (18%)]. The rates of SWL despite some fluctuations seem to have steadily decreased over the study period (*p* = 0.2) (Fig. 3), although there was a clear fall in the number of non-English articles published over time. When comparing the two time periods, there were a total of 486 articles published in period-1, which had decreased by almost 20% to 401 articles in period-2 (*p* = 0.18). The number of English/non-English language articles in period 1 and period 2 was 384/102 and 345/56 articles, respectively, suggesting a drop of 10 and 45% for English and non-English articles during period-2.

#### Open surgery, pyelolithotomy, and laparoscopic surgery

**Fig. 5:**
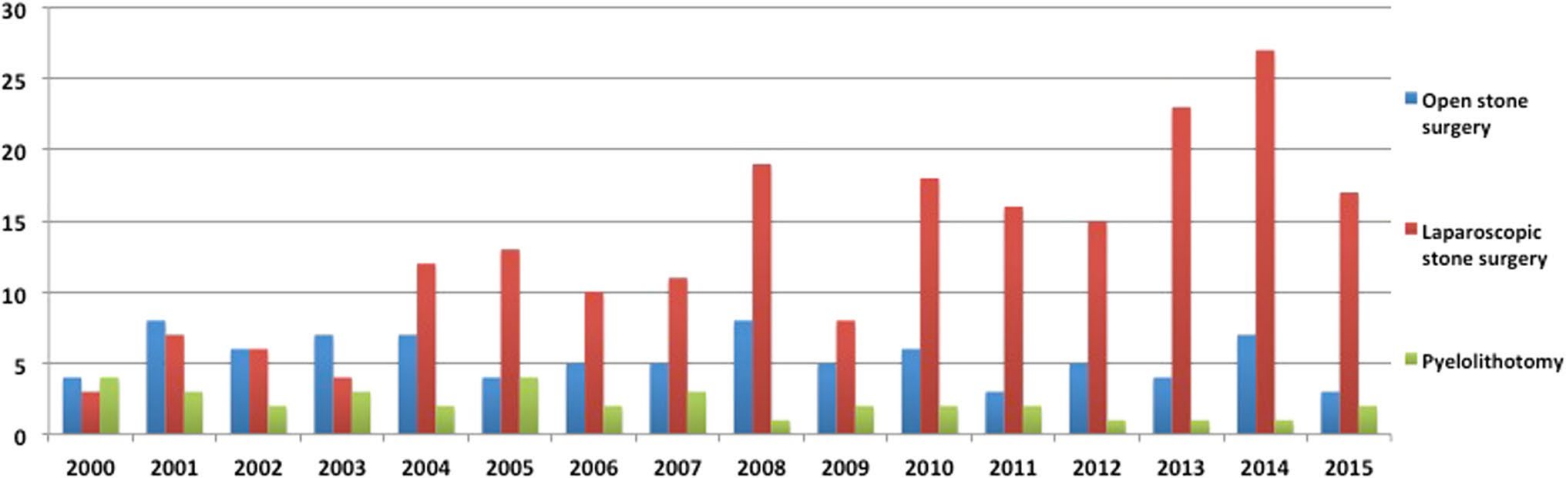
Publication trends for uncommon interventions (Open stone surgery, laparoscopic stone surgery and pyelolithotomy)

During the last 16 years, a total of 331 papers have been published on these interventions for Urolithiasis, including open stone surgery (*n* = 87, 26%), laparoscopic stone surgery (*n* = 209, 63%), and pyelolithotomy (*n* = 35, 11%) (Fig. 5). While there was a steady decline for open stone surgery and pyelolithotomy, there was a steep rise in laparoscopic stone surgery.

When comparing the two time periods, there were 135 intervention papers in period-1, which had increased to 196 intervention papers in period 2 (Table 1). The increase was seen only for laparoscopic surgery (+116%, *p* < 0.001), while it decreased for open surgery (-11%, *p* = 0.17) and pyelolithotomy (−47%, *p* = 0.003).

### Use of simulation in endourology

**Fig. 6:**
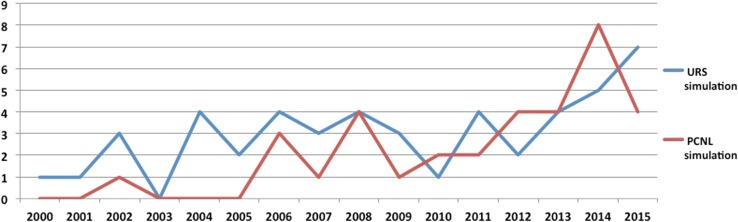
Publication trends for Ureteroscopy and PCNL Simulation

During the last 16 years, a total of 82 papers have been published on simulation for stone surgery including 48 papers for URS and 34 papers for PCNL. There seems to have been a steady rise in simulation-based papers over the last 16 years (Fig. 6).

When comparing the two time periods, there were 23 papers published in period 1, which had more than doubled (increase of 159%) to 59 papers in period 2. While URS-based simulation papers had increased from 18 to 30 during this period (67% rise, *p* = 0.007), PCNL-based simulation papers had increased from 5 to 29 (a rise of 480%, *p* < 0.001). There was only one simulation-based PCNL paper published in the first 6 years of the study period (2000–2005), with a linear increase since then, potentially showing a renewed interest in this technique with minimally invasive PCNL techniques.

### Laser technology in endourology

**Fig. 7:**
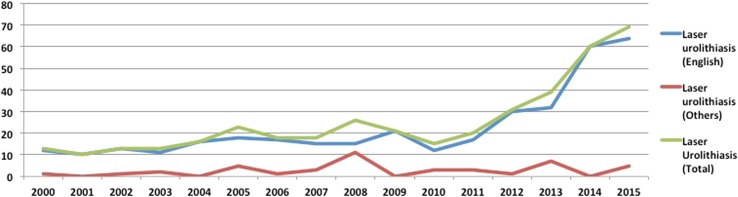
Publication trends for use of lasers in urolithiasis

During the last 16 years, a total of 406 papers have been published on lasers for stone surgery, including 363 (89%) English language and 43 mon-English language articles (Fig. 7). There seems to be a steep rise in the articles published since 2010 (*p* < 0.001).

When comparing the two time periods, there were 124 papers published in period-1, which had more than doubled (increase of 126%) to 281 papers in period 2 (*p* < 0.02). The number of English/non-English language articles in period 1 and period 2 was 112/13 and 251/30 articles, respectively, suggesting a rise of around 125 and 130% in the study period.

## Discussion

### Meaning of our study

This is one of the first bibliometric studies in the field of ‘Urolithiasis’ looking at publication trend over the last 16 years (2000–2015). There is a significant rise in the number of published papers (fourfold), with a rising trend in most interventions for stone disease. Over the period as a whole, the number of papers analyzing stone surgery procedures witnessed dramatic changes, with the main features experiencing a significant increase in URS and PCNL, a drastic decline in open surgery and pyelolithotomy, and a gradual decrease in SWL. As can be seen from the global trend graph of interventions, at the mid term of this research period minimally invasive techniques such as URS and PCNL overtook SWL, which used to predominate at the beginning of the period. This is partly explained by the innovations in technology, which resulted in improvement and innovation in flexible ureteroscopes with digital and now disposable scopes. A similar transformation has been seen with PCNL with miniaturization of this technique with newer more minimally invasive kits and techniques.

Besides intervention, our review also shows that published papers on simulation have increased over the last two decades showing not only a renewed interest but a realization that simulators have a huge role in the modern day Endourology training and a new generation of simulators that provide ‘realism’ in training [7, 8]. Similarly, the trend in the use of lasers mirrors the progress in the field of laser technology [9], which leads to an increase in effectiveness without a corresponding growth of invasiveness and complication rate.

### Strengths and weakness of bibliometric trend analysis

We used PubMed for bibliometric analysis of trends on ‘Urolithiasis’ over the last 16 years as it gives a more realistic view of research trends in Endourology [10]. The results reflect a global and extensive rise in the publications in this area. Although PubMed is an excellent source for bibliometric analysis compared to Scopus or Web of Science, there are some journals, which may not be indexed on PubMed [11]. Given the limitations to our review and the fact that typical bibliometric analysis are biased toward English language articles, to make it more inclusive, we also included all Non-English language articles that had an English abstract in PubMed. However, articles with native language abstract or no abstract were excluded. Similarly a citation index or detailed co-author affiliation could not be established from PubMed, which might have provided more insight in these publication trends.

The design of the current study permits a worldwide assessment of publication trends on urolithiasis along with all variety of interventions for it. The number of published papers in the second half (period-2) has increased significantly. Although pyelolithotomy was done either as open or laparoscopic surgery, as this was not clear on published papers and often included both these techniques, we analyzed it separately. While innovations in minimally invasive surgery have led to a decline in open surgery, it has seen a rise in URS and PCNL. It also highlights a growing popularity of the modern minimally invasive PCNL techniques for stone disease, such as Mini, Ultra-mini, and Micro PCNL that uses holmium laser for stone fragmentation [12]. Similarly, the use of URS has risen due to its feasibility and effectiveness in complex patients and difficult stones, including stones in solitary kidneys, bleeding diathesis, pediatrics, and pregnancy, for which a more invasive alternative treatment option would expose patients to significantly higher risks of major complications [13, 14].

A further explanation of this increase in ‘surgical’ papers could be the ‘quick’ stone-free rate achieved with surgery compared to SWL without increasing dramatically the complication rate [3]. Furthermore, the advances in SWL technology have not been so prominent compared to PCNL and URS. Therefore, although the ‘publication’ trend is in favor of the invasive procedures, we do not imply that urologists either ‘prefer’ or ‘use’ more the invasive techniques compared to SWL. Quantification of published data gives insight into areas of intervention and articles on newer techniques are more likely to be published which was also evident in the growth of minimally invasive PCNL techniques (Table 2). Similarly, training and increase in local and international fellowships have also ‘popularized’ invasive techniques [15].

**Table 2:** Rise of minimally invasive PCNL papers (Mini, Micro, and Ultra-mini PCNLs)

Years	PNL, percutaneous nephrolithotomy Total articles—(English + non-English)	Mini PCNL	Micro PCNL	Ultra-mini PCNL
2000	18	2		
2001	26	2		
2002	29	1	1	
2003	33	1	1	
2004	38	1		
2005	56	3		
2006	67	2		
2007	91	9		
2008	143	10		
2009	121	3		
2010	123	4		
2011	173	10	1	
2012	211	15	1	
2013	282	21	6	4
2014	209	12	10	1
2015	195	26	9	7
Total	1715	122	27	12

Although this review reflects that publication rate in ‘Interventions for Urolithiasis’ has changed, this may not entirely reflect the individual interventions offered/performed by clinicians. Future study analyzing the citation index, country, or institution of origin of the study and use of multiple databases might be useful for a more comprehensive study. This might also help in gathering the landmark papers in endourology or in identifying self-citations and potential articles which were most cited in the literature.

## Conclusions

Published papers on intervention for Urolithiasis have risen over the last 16 years. While there has been a steep rise of URS and minimally invasive PCNL techniques, SWL and open surgery have shown a slight decline over this period. A similar increase has also been seen for the use of simulation and lasers in Endourology. The results of our study might be useful in future resource planning for primary and secondary care interventions for urolithiasis.
